# Interaction between SCO-spondin and low density lipoproteins from embryonic cerebrospinal fluid modulates their roles in early neurogenesis

**DOI:** 10.3389/fnana.2015.00072

**Published:** 2015-05-28

**Authors:** América Vera, Antonia Recabal, Natalia Saldivia, Karen Stanic, Marcela Torrejón, Hernán Montecinos, Teresa Caprile

**Affiliations:** ^1^Department of Cell Biology, Faculty of Biological Sciences, University of ConcepciónConcepción, Chile; ^2^Faculty of Biological Sciences, Department of Biochemistry and Molecular Biology, University of ConcepciónConcepción, Chile

**Keywords:** embryonic cerebrospinal fluid, SCO-spondin, low density lipoproteins, chick embryo, neurogenesis, brain development

## Abstract

During early stages of development, encephalic vesicles are composed by a layer of neuroepithelial cells surrounding a central cavity filled with embryonic cerebrospinal fluid (eCSF). This fluid contains several morphogens that regulate proliferation and differentiation of neuroepithelial cells. One of these neurogenic factors is SCO-spondin, a giant protein secreted to the eCSF from early stages of development. Inhibition of this protein *in vivo* or *in vitro* drastically decreases the neurodifferentiation process. Other important neurogenic factors of the eCSF are low density lipoproteins (LDL), the depletion of which generates a 60% decrease in mesencephalic explant neurodifferentiation. The presence of several LDL receptor class A (LDLrA) domains (responsible for LDL binding in other proteins) in the SCO-spondin sequence suggests a possible interaction between both molecules. This possibility was analyzed using three different experimental approaches: (1) Bioinformatics analyses of the SCO-spondin region, that contains eight LDLrA domains in tandem, and of comparisons with the LDL receptor consensus sequence; (2) Analysis of the physical interactions of both molecules through immunohistochemical colocalization in embryonic chick brains and through the immunoprecipitation of LDL with anti-SCO-spondin antibodies; and (3) Analysis of functional interactions during the neurodifferentiation process when these molecules were added to a culture medium of mesencephalic explants. The results revealed that LDL and SCO-spondin interact to form a complex that diminishes the neurogenic capacities that both molecules have separately. Our work suggests that the eCSF is an active signaling center with a complex regulation system that allows for correct brain development.

## Introduction

In all vertebrates the central nervous system (CNS) originates from the neural tube, a hollow structure delimited by neuroepithelial cells and filled with embryonic cerebrospinal fluid (eCSF). Correct brain development requires interaction between the neuroepithelium and eCSF. Through this interaction, the neuroepithelium survives, proliferates, and differentiates in response to molecules present in the eCSF. Some of these molecules originate in the serum, while others are secreted by neuroepithelial cells (Gato et al., 2005; Gato and Desmond, 2009).

Embryonic cerebrospinal fluid participates in CNS development through two mechanisms. First, the accumulation of eCSF in brain cavities generates an intraluminal pressure that promotes the proliferation of neuroepithelial cells (Desmond and Jacobson, 1977; Desmond et al., 2005, 2014). Second, eCSF contains growth factors and morphogens that interact with the neuroepithelium. Different studies in chick (*Gallus gallus*) and rat (*Rattus norvegicus*) embryos found eCSF to promote neural cell differentiation, proliferation, and survival in neuroepithelium explants, although this was dependent on the embryonic stage of the eCSF and of the explants analyzed (Lehtinen et al., 2011; Yari et al., 2013; Gato et al., 2014). These studies showed that the eCSF has molecules which provide an essential environment for neuroepithelial cells, as compared to serum and serum-free mediums. It is additionally implied that some eCSF molecules are of non-plasmatic origin and would be directly secreted by neuroepithelial cells (Gato et al., 2005; Salehi and Mashayekhi, 2006; Gato and Desmond, 2009; Martin et al., 2009; Zappaterra and Lehtinen, 2012; Vera et al., 2013).

The main constituents of eCSF are proteins whose enrichment is several folds higher during embryonic stages than in the adult cerebrospinal fluid (Birge et al., 1974; Dziegielewska et al., 1980). Proteomic analysis of eCSF revealed the presence of several factors related to cell differentiation or proliferation, such as bone morphogenetic proteins (Lehtinen et al., 2011; Segklia et al., 2012), lipoproteins (Parada et al., 2008a), the fibroblast growth factor (Martin et al., 2006), the insulin growth factor (Salehi et al., 2009; Lehtinen et al., 2011), the nerve growth factor (Mashayekhi et al., 2009), sonic hedgehog (Hh; Huang et al., 2010b), Wingless family proteins (Lehtinen et al., 2011; Johansson et al., 2013), SCO-spondin (Vio et al., 2008; Vera et al., 2013), and retinoic acid (Alonso et al., 2011) and associated molecules such as its precursor, all-trans-retinol, and its carrier, the retinol binding protein (Parada et al., 2008b). The roles for some of these molecules have been described *in vitro* using explants of neuroepithelial cells (Parada et al., 2008a; Vera et al., 2013), with findings that lipoproteins and SCO-spondin have important functions in neurodifferentiation.

Lipoproteins are water-soluble macromolecular carriers that facilitate the delivery of lipid cargo into target cells. Different lipoproteins are present in chick eCSF, especially between stages HH20 to HH27, when maximum neuronal differentiation of neuroprogenitor cells occurs. At these stages, the main lipoproteins are low-density lipoproteins (LDL) and very low density lipoproteins (VLDL; Bachy et al., 2008; Parada et al., 2008a). The presence of Apolipoprotein B (ApoB), the principal proteic fraction of LDL, in the eCSF has been described in the mouse and chicks, and its relevance during brain development was revealed in ApoB-mutated or -knockout (KO) mice that died at an early developmental stage and exhibited severely impaired brain developments, such as exencephaly and hydrocephaly (Homanics et al., 1993; Farese et al., 1995).

*In vitro* experiments have shown that LDL is critical during early stages and promotes the neurodifferentiation and proliferation of neuroepithelial cells. In fact, Parada et al. (2008a) described that LDL is by itself capable of generating the same neurodifferentiation in mesencephalic explants as complete eCSF. However, adding LDL together with an eCSF fraction depleted of lipoproteins to the culture medium decreased neurodifferentiation by 60%. This result suggests that LDL is a potent neurogenic factor regulated by proteins present in the eCSF.

SCO-spondin is a glycoprotein of the thrombospondin family with a high molecular weight (Gobron et al., 1996; Didier et al., 2007). SCO-spondin is secreted by the diencephalic roof plate from early stages of development, both basally, into the extracellular matrix (ECM), where it comes into contact with posterior commissure axons, and apically, into the eCSF, where it can be found as a soluble or aggregated (forming Reissner’s fiber) molecule (Schoebitz et al., 1986; Rodríguez et al., 1998; Caprile et al., 2009; Stanic et al., 2010; Vera et al., 2013).

Regarding its functions, *in vivo* analysis using shRNA to knockdown SCO-spondin in *G. gallus* embryos showed increased neuroepithelium proliferation and decreased differentiation, together with diencephalon and mesencephalon hyperplasia and other morphological defects on the posterior commissure and pineal gland. *In vitro* experiments confirmed the importance of eCSF-derived SCO-spondin, where mesencephalic explants cultured in the presence of eCSF abruptly diminished neurodifferentiation but increased the number of undifferentiated cells after the addition of SCO-spondin antibodies. Together, these results indicate that SCO-spondin is essential for embryonic development and that it regulates the balance between neuroepithelial proliferation and differentiation (Vera et al., 2013).

SCO-spondin is a giant protein with multidomain organization that includes, among others, 26 thrombospondin type 1 repeats (TSR; implicated in protein-protein interactions) and eight contiguous LDL receptor class A domains (LDLrA; Didier et al., 2007). Within the members of the LDL receptor family, the LDLrA domain is also repeated in tandem and comprizes the protein region responsible for LDL binding proprieties (Russell et al., 1989; Yamamoto and Ryan, 2009). The presence of both molecules in the eCSF, SCO-spondin with LDLrA domains and LDL, allow us to suggest that SCO-spondin could be binding LDL, and probably others molecules, thereby controlling the effects of neurodifferentiation.

The present work provides evidence that LDL and SCO-spondin from the eCSF form a complex, and that this interaction is important in modulating the neuroepithelium differentiation generated by both molecules.

## Material and Methods

### Chick Embryos

Fertilized chick eggs were incubated at 38°C in a humidified incubator for specific time intervals. Embryos were staged according to Hamburger and Hamilton (1992). Experiments were conducted following the guidelines outlined in the Biosafety and Bioethics Manual of the National Commission of Scientific and Technological Research (CONICYT, Chilean Government) and the Ethics Committee of the University of Concepción.

### eCSF Extraction

Embryonic cerebrospinal fluid from HH23 and HH30 embryos was obtained as previously described (Gato et al., 2005) with slight modifications. In order to avoid contamination with neuroepithelial cells, eCSF was gently sucked up under the dissecting microscope with a glass micro-needle that was carefully introduced into the middle of the mesencephalic cavity. To minimize protein degradation, eCSF samples were kept at −15°C with a protease inhibitor cocktail (Sigma P2714), aliquoted, and frozen at −80°C until use.

### Organotypic Cultures of Mesencephalic Neuroectoderm

Organotypic cultures of optic tectum were performed as described by Gato et al. (2005) and maintained at 37°C with 5% CO_2_ for 24 h in the presence of 0.01 mM 5-Bromo-2′-deoxyuridine (BrdU; Sigma) and one of the six following media: (i) Dulbecco’s Modified Eagle’s medium (DMEM; Sigma); (ii) Conditioned medium obtained from the supernatant of HH36 subcommissural organ (SCO) culture maintained for 4 days in DMEM. The presence of SCO-spondin in this medium was confirmed by Western blot (Vera et al., 2013); (iii) Conditioned medium containing SCO-spondin plus anti-SCO-spondin (1:300); (iv) 0.02 μg/μl of LDL in DMEM; (v) 0.02 μg/μl of LDL in conditioned medium containing SCO-spondin; or (vi) the same as medium (v), but with the addition of anti-SCO-spondin (1:300). After 24 h, the explants were fixed in 4% paraformaldehyde for 20 min, dehydrated in ascending concentrations of alcohols, and embedded in Paraplast. The explants were oriented to obtain 5–7 μm thick frontal sections of the mesencephalon explant. Sections were immunostained with mouse monoclonal primary antibodies raised against anti-BrdU (G3G4, Developmental Studies Hybridoma Bank, University of Iowa, Iowa City, IA) and anti-βIII tubulin (clone Tuj1, Promega, Madison, WI, USA) antibodies.

Antibodies were diluted in a Tris-HCl buffer containing 1% bovine serum albumin (Tris-BSA). Alexa Fluor 546 Goat anti-mouse (Invitrogen, Carlsbad, CA, USA) was diluted to 1:100 in Tris-BSA and incubated for 2 h at room temperature. Nuclei were visualized with TO-PRO-3 (Invitrogen, Carlsbad, CA, USA). Images were acquired with a laser confocal Nikon Eclipse TE2000-U microscope. The proliferation process was measured by analyzing the quantity of BrdU positive nuclei vs. the total explant area, while the process of neurodifferentiation was measured by the area stained with tubulin βIII vs. the total explant area. These analyses were performed with the ImageJ program by measuring the area of the explant, followed by transforming the image into binary and counting the number of nuclei or the immunopositive area. Each condition was repeated in triplicate, and statistical analyses were performed using the Student’s *t* test, differences were considered significant at *p* < 0.05.

### Agarose Gel and Sudan Black Staining of Lipids

20 μl of eCSF from HH23 embryos were stained at 4°C overnight with 10 μl of 1% Sudan black previously diluted in 30% ethanol. Following this, the samples were loaded onto 0.8% agarose gel with 0.05 M barbital buffer pH 8.6, thereby allowing visualization of lipid components as black bands. Afterwards, the gel was electrotransferred onto a nitrocellulose membrane in a buffer containing 25 mM TRIS-HCl pH 8.3, 192 mM glycine, 0.2% SDS, and 20% methanol, at 100 mA for 5 h. Nonspecific protein binding sites were blocked by incubating the nitrocellulose membranes with 5% nonfat milk in a 0.1 M phosphate saline buffer containing 0.1% Tween-20 for 2 h at room temperature. The lipid bands maintained their black color stain after transference, allowing for the identification of the different lipoproteins. As lipoprotein controls, commercial human LDL (Sigma L8292), human HDL (kindly donated by C. Radojkovicby; González-Pecchi et al., 2015), and fetal bovine serum were used.

Membranes were probed overnight with a rabbit anti-Reissner’s fiber glycoproteins (AFRU) antibody (1:5000) that recognizes SCO-spondin (Caprile et al., 2009), followed by incubation with anti-IgG rabbit secondary antibody (1:5000; Jackson Immunoresearch) for 2 h at room temperature. For Western blots of the immunoprecipitates, SCO-spondin was detected through the anti-rat SCO-spondin antibody and anti-IgG rat secondary antibody (1:5000; Jackson Immunoresearch). Immunoreactive proteins were detected with an enhanced chemiluminescence system (SuperSignal, Pierce) according to the manufacturer’s instructions.

### Coimmunoprecipitation

Immunoprecipitation was performed using the Pierce Classic IP Kit (Thermo Scientific, 26146) following the manufacturer’s instructions. The column for immunoprecipitation was prepared with 50 μl of protein A agarose, which was washed three times with 100 μl wash buffer (0.025 M Tris, 0.15 M NaCl, 0.001 M EDTA, 1% NP-40, 5% glycerol, pH 7.4) and centrifuged at 4°C and 1000 *g* for 1 min. The eCSF of HH23 embryos incubated with anti-SCO-spondin (1:100) at 4°C overnight was added to the column and incubated at 4°C for 3 h with agitation, followed by centrifugation at 1000 *g* for 1 min. The column was then washed three times with 200 μl of Tris buffer followed by centrifugation at 1000 *g* for 1 min. Finally, elution was performed with the elution buffer contained in the Pierce Classic IP Kit followed by centrifugation at 1000 *g* for 1 min. This elution was repeated five times. The eluates were stained with Sudan black and loaded onto a 0.8% agarose gel with 0.05 M barbital buffer pH 8.6 in order to analyze the presence of lipoproteins in the immunoprecipitate.

### Immunohistochemistry

For colocalization of ApoB and SCO-spondin in the mesencephalon, brain tissue of HH24 embryos was fixed in 4% paraformaldehyde for 12 h and processed following the same protocols described for mesencephalic explants. The brain was oriented in order to obtain sagittal sections. The antibodies used were anti-ApoB (1:100; Bustos et al., 1998) and anti-SCO-spondin (1:5000; Caprile et al., 2009), while vimentin (1:1; H5, Developmental Studies Hybridoma Bank, University of Iowa, Iowa City, IA) was used as a control.

### Bioinformatics Analyses

Bioinformatics analyses were performed for the region of SCO-spondin (Accession: NP_001006351.2; GI: 110082727) that contains eight LDLrA domains arranged in tandem. This region is located between amino acids 1363 and 1740 and contains 377 amino acids.

The primary structures of these domains were compared to one another and with the LDLrA domains contained in the human (*Homo sapien*s) LDL receptor sequence (Accession: NP_000518.1; GI: 4504975) using the Clustal Omega program.

The hydrophobicity of this SCO-spondin fragment was analyzed using the Kyte and Doolittle ProtParam algorithm supplied by the ExPASy proteomic tool^1^ with an open window of nine amino acids.

The Neff-PPAS algorithm predicted the tertiary structure, and stereochemistry validation of the model was performed with RAMPAGE^2^ and visualized with PyMol v.0.98.^3^ The obtained structure model was validated by analyzing the phi (φ) and psi (ψ) torsion angles and determined using a Ramachandran plot.

## Results

The possible interaction between SCO-spondin and LDL was analyzed by three different experimental approaches: (1) Bioinformatics analyses of the SCO-spondin region that contains eight LDLrA domains in tandem and of comparisons with the LDL receptor consensus sequence; (2) Analysis of the physical interactions of both molecules through colocalization in native agarose gels, using immunohistochemical colocalization in embryonic chick brain, and through the coimmunoprecipitation of LDL with SCO-spondin antibodies; and (3) Analysis of functional interactions when these molecules were added to a culture medium of mesencephalic explants.

### Bioinformatics Analyses of SCO-Spondin

**Figure 1:**
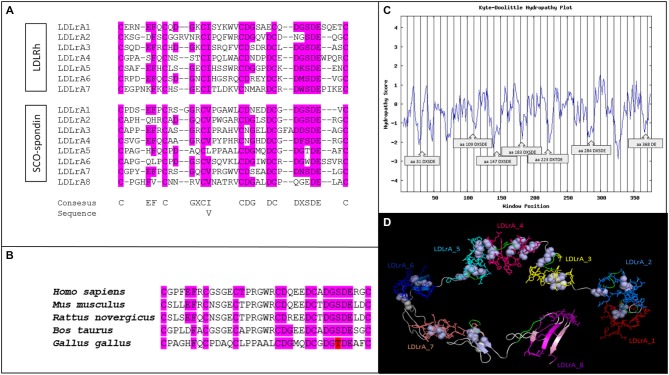
**Bioinformatics analyses of LDLrA domains present in the SCO-spondin sequence of *Gallus gallus*. (A)** Multiple alignment of SCO-spondin LDLrA domains and comparison with the LDLrA domain sequence of the human LDL receptor (LDLRh). **(B)** Multiple alignment of the SCO-spondin LDLrA5 domain from different species. For all species analyzed this domain conserved the DxSDE motif, or a conservative substitution of serine to threonine (in red). **(C)** Hydropathic plot of the SCO-spondin sequence that contains the LDLrA domains, showing its hydrophilic character, especially for the regions containing the DxSDE motifs. **(D)** Tertiary structure of the SCO-spondin region that contains the LDLrA domains arranged in tandem. Each domain is shown in a different color; DxSDE motifs are highlighted in green, and disulfide bridges are in white.

The SCO-spondin of *G. gallus* (Accession: NP_001006351.2; GI: 110082727) is a giant protein of 5225 amino acids that contains several LDLrA domains. In order to perform bioinformatics analyses, the region contained between amino acids 1363 and 1740 was selected because it contains a cluster of eight LDLrA domains. The sequences of these domains were compared using Clustal Omega with the seven LDLrA domains present in the human LDL receptor (Figure 1A). The results showed a total conservation of the six structurally important cysteines as well as a high conservation of the DxSDE motif, which is important for the Ca^++^ and LDL binding described in all the members of the LDLr family (Daly et al., 1995; Jeon and Blacklow, 2005; Guttman et al., 2010). In summary, the LDLrA domains present in SCO-spondin contained the consensus sequence important to maintaining domain stability and the ligand binding proprieties of LDL receptor family members.

Previous studies on the LDL binding capacities of human LDLr demonstrated that a cluster of LDL motifs is necessary, but only the central LDLrA motifs are crucial for LDL binding (Fisher et al., 2004). In this regard, SCO-spondin LDLrA4 maintained the DxSDE consensus sequence, and in LDLrA5 there was a conserved serine to threonine substitution, an amino acid with equivalent features. Several LDL motifs were conserved in all of the reported SCO-spondin sequences, but there are no reports about the conservation of the amino acids important for LDL binding. For this reason the LDLrA5 domains of *H. sapiens* (Accession: NP_940857.2; GI: 134031945), *Bos taurus* (Accession: NP_777131.1; GI: 28195400), *Mus musculus* (Accession: CAD42654.1; GI: 27527438), and *Rattus novergicus* (Accession: NP_001007017.1; GI: 55741770) were analyzed. The results showed that all of these conserved the six cysteines and the DxS/TDE motif important for LDL binding (Figure 1B).

The hydropathic characteristics of the SCO-spondin fragment containing the LDL domains were plotted using the ExPASy Protscale tool. The hydropathic index of the entire fragment was −0.406 Kj/mol. This suggests that, due to its hydrophilic character, this fragment would be on the SCO-spondin surface and exposed to eCSF. A detailed analysis of the Kyte and Doolittle Hydropathy Plot revealed that the most hydrophilic peaks corresponded to DxSDE motifs (Figure 1C).

The tertiary structure of the SCO-spondin fragment was predicted with the Neff-PPAS program, available in the LOMETS server, using 1n7d (extracellular region of human LDLr) as a template. The model was visualized using PyMol v.0.98 (Figure 1D).

Validation of the obtained structural model was performed by inspecting the backbone conformation through analyzing the phi (φ) and psi (ψ) torsion angles. The predicted conformation was a stable structure with 278 amino acids in favored positions (75.9%), 64 amino acids in allowed positions (17.3%), and 25 amino acids in atypical positions (6.8%).

An important feature of the predicted tertiary structure was that the LDLrA domains surrounded a central pocket that in other members of the LDL receptor family has been proposed as the region occupied by the LDL molecule (Huang et al., 2010a). Considering this, the tertiary structure prediction of this study showed that DxSDE motifs (Figure 1D, in green) were exposed to this central pocket, especially in the central LDLrA3, 4, 5, and 7 domains. As stated before, in other members of the LDL receptor family, these central LDLrA domains are the most relevant for LDL binding (Fisher et al., 2004).

### Physical Interaction Between LDL and SCO-Spondin

**Figure 2:**
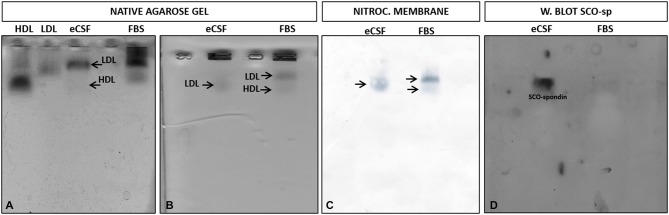
**Migration pattern of lipoproteins and SCO-spondin from the eCSF. (A)** Detection of eCSF lipoproteins by staining with Sudan black and posterior native agarose gel electrophoresis, showing the presence of low density lipoproteins (LDL) and high density lipoproteins (HDL) (arrows) in the eCSF of HH30 chick embryo. Human LDL and HDL and fetal bovine serum (FBS) were used as positive controls. **(B–D)** Migration pattern of lipoproteins and SCO-spondin present in the eCSF. **(B)** Native agarose gel electrophoresis stained with Sudan black (lipid stain), revealing the presence of lipoproteins (arrows) in the eCSF and fetal bovine serum (FBS; control). **(C)** Transference of the gel shown in **(B)** to nitrocellulose membrane. **(D)** Western blot with anti-SCO-spondin of the nitrocellulose membrane shown in **(C)**, revealing a migration pattern of SCO-spondin identical to the eCSF LDL stained with Sudan black **(B,C)**.

In order to study the LDL/SCO-spondin interaction, a protocol for lipoprotein detection was previously standardized based on Parada et al. (2008a). Thus, eCSF was incubated with Sudan black, a lipophilic stain, which was followed by native agarose gel electrophoresis. As controls, commercial human LDL, HDL purified from human serum (González-Pecchi et al., 2015), and fetal bovine serum were used. The results showed that at early stages of development, the eCSF of chick embryos contained different lipoproteins, with LDL being the most evident (Figure 2A). As expected, the LDL and HDL of the eCSF did not have a migration pattern identical to human LDL and HDL, since these lipoproteins have different sizes depending on age and gender (McNamara et al., 1987).

As a first approximation to study the possible interaction between LDL and SCO-spondin, a migration analysis of both molecules was performed in native agarose gels. For this, eCSF from HH30 chick embryos was incubated with Sudan black, followed by native agarose gel electrophoresis (Figure 2B) and electrotransference to a nitrocellulose membrane controlled by nitrocellulose black staining (Figure 2C). The membrane was then incubated with anti-SCO-spondin for Western blot analysis (Figure 2D). In order to better separate the different components of the eCSF, the running time of electrophoresis was increased, although this generated some spreading of lipoproteins in the gel (compare Figures 2A,B). The results showed that LDL and SCO-spondin from chick eCSF had an identical migration pattern in native agarose gels, suggesting a possible interaction between these molecules.

**Figure 3:**
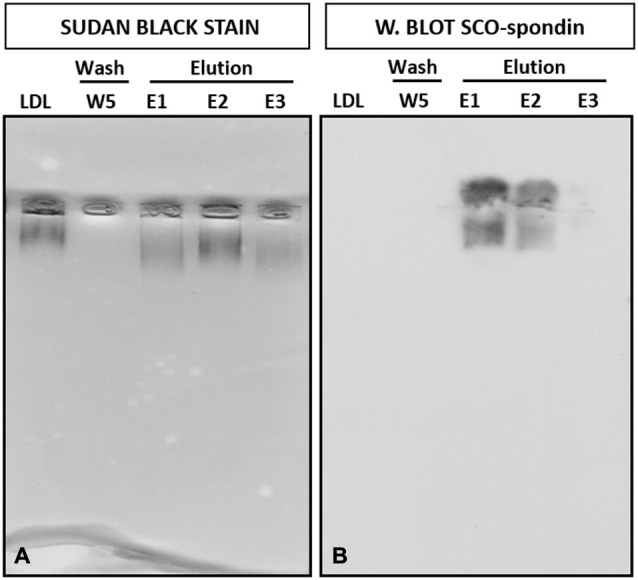
**Coimmunoprecipitation of LDL and SCO-spondin from the eCSF. (A)** Native agarose gel electrophoresis of eCSF from HH23 chick embryo immunoprecipitated with SCO-spondin antibodies. Lane 1: LDL commercial. Lane 2: W5: last wash of the immunoprecipitate, revealing the absence of lipoproteins. Lane 3–5: Elution fractions of the immunoprecipitate, showing the presence of LDL. **(B)** Western blot with anti-SCO-spondin of the gel shown in **(A)**, revealing the presence of this protein in the eluted fractions.

The physical interaction between SCO-spondin and LDL was analyzed by coimmunoprecipitation assays. For this, the eCSF from HH23 embryos was previously incubated for 4 h with 0.001 μg/μl of commercial LDL followed by immunoprecipitation with anti-SCO-spondin antibodies. The immunoprecipitate, as well as the last wash, were stained with Sudan black, followed by agarose gel electrophoresis, which showed that LDL was present in the immuprecipitate (Figure 3A). The gel was then electrotransferred in order to check if SCO-spondin was also in the immunoprecipitate. The presence of this protein was found in the same elution aliquots as LDL (Figure 3B). This result suggest that both proteins form a complex in the eCSF. Note that after the elution (pH 2.8) the molecular interactions are broken, including the LDL/SCO-spondin complex, and the isoforms of SCO-spondin migrated differently than in the Figure 2D.

**Figure 4:**
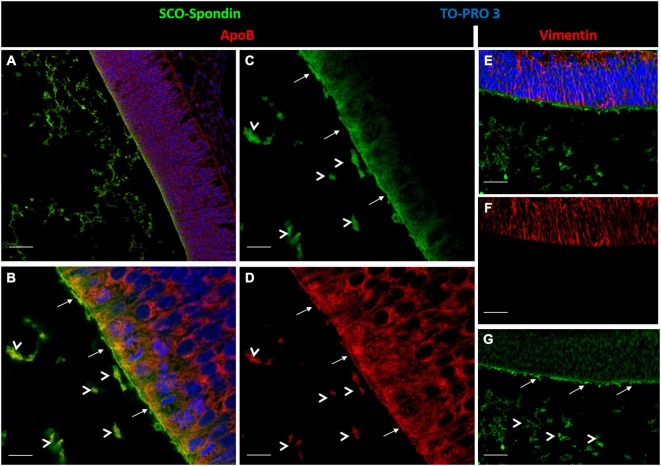
**Inmunohistochemical localization of ApoB and SCO-spondin. (A)** Sagittal sections of dorsal mesencephalon of HH24 chicken embryos. Immunohistochemistry with antibodies against SCO-spondin (green) and ApoB (red) counterstained for nuclei with TO-PRO-3 (blue). **(B–D)** Higher magnification of **(A)**, showing strong immunoreactivity for SCO-spondin **(C)** and ApoB **(D)** in the apical region of neuroepithelial cells (arrows). Arrowheads show particles in the encephalic cavities positive for both antibodies. **(E–G)** Immunohistochemistry with anti-vimentin (red) as a negative control. Scale of bars is 50 μm in **(A, E–G)**; 10 μm in **(B–D)**.

The formation of this LDL/SCO-spondin complex was also studied by double immunohistochemistry using anti-SCO-spondin and anti-ApoB (the main proteic fraction of LDL) antibodies. Immunohistochemical analysis of HH24 chick brain revealed immunoreactivity for both antibodies on the apical region of neuroepithelial mesencephalic cells (arrows in Figures 4A–D), as well as in eCSF particles present in the encephalic cavities (arrowheads in Figures 4A–D). As a negative control, the location of vimentin, an intermediate filament, was analyzed, finding that it was not present in either eCSF particles or in the apical region of the neuroepithelial cells (Figures 4E–G).

### Functional Interaction Between LDL and SCO-Spondin

**Figure 5:**
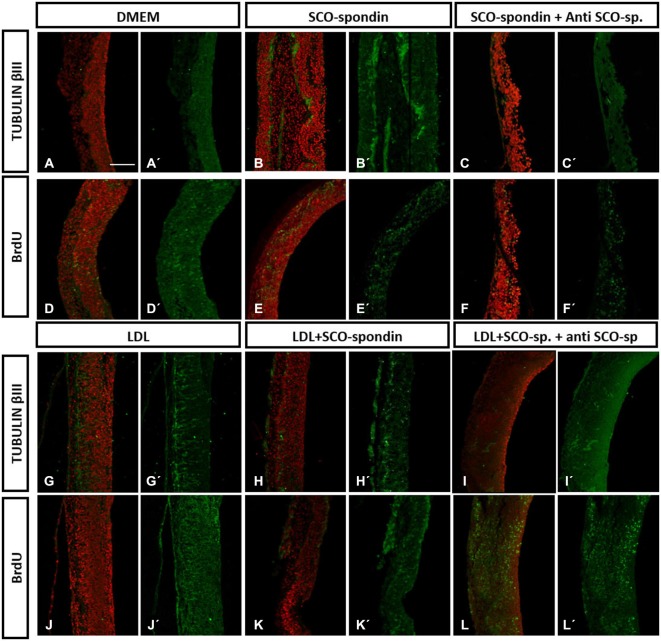
**Immunohistochemistry of mesencephalic neuroepithelial explants under various experimental conditions**. Optic tectum explants from HH20 embryos cultured for 24 h in the presence of: (i) DMEM as a negative control **(A,D)**; (ii) Conditioned medium from SCO culture explants **(B,E)**; (iii) Conditioned medium from SCO culture explants plus anti-SCO-spondin antibodies **(C,F)**; (iv) DMEM in the presence of LDL **(G,J)**; (v) Conditioned medium from SCO culture explants in the presence of LDL **(H,K)**; and (vi) The same as (v), but with anti-SCO-Spondin **(I,L**). The explants were analyzed for the presence of βIII tubulin **(A–C, G–I)** and BrdU incorporation **(D–F, J–L)**. Panels **(A′–L′)** show the merger, with the TO-PRO-3 nuclear signal used to counterstain the tissue in red. Scale bar in A is 50 μm, all the images have the same magnification.

**Figure 6:**
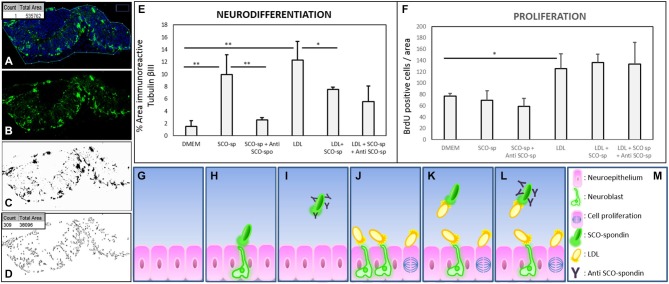
**Quantitative analysis of proliferation and neurodifferentiation in mesencephalic explants under various experimental conditions. (A–D)** Steps performed to quantify cell proliferation and differentiation using the ImageJ program. **(A)** The explants were enclosed and the total area measured (box in A). **(B–C)** The image of the immunostained explant (without TOPRO-3) was transformed to a binary image. **(D)** The total number of particles from the binary image were measured (count in box D), and the area occupied by these particles was summarized (total area in box D). When the analyzed figure was immunostained with anti-BrdU, the count represented the number of positive nuclei. When the analyzed figure was immunostained with anti-tubulin βIII, the total area represented the magnitude of neurodifferentiation. **(E)** Quantitative analysis of neuroepithelial cells undergoing neural differentiation, as measured by the % of area positive for tubulin βIII. **(F)** Quantitative analysis of cell proliferation was measured by the number of BrdU-positive cells per area. **(G–M)** Scheme showing the interpretation of proliferation and neurodifferentiation analyses. The results showed that SCO-spondin by itself was able to promote the neurodifferentiation of neuroepithelial cells **(E,H)**, but this capacity drastically diminished in presence of anti-SCO-spondin **(E,I)** or LDL **(E,K)**. On the other hand, LDL by itself was able to promote neurodifferentiation **(E,J)** and proliferation **(F,J)**. The proliferation generated by LDL was independent of the presence of SCO-spondin and anti-SCO-spondin, but, rather, neurodifferentiation decayed in the presence of SCO-spondin (with or without anti-SCO-spondin), suggesting that the LDL-SCO-spondin complex is unable to promote neurodifferentiation. Statistical analyses were performed using the Student’s t test, *p < 0.05; **p < 0.01. Bars represent the mean ± SEM of three experiments.

The capacity of LDL and SCO-spondin to increase neurodifferentiation in embryonic mesencephalic explants when separately added to the culture medium was previously reported (Parada et al., 2008a; Vera et al., 2013). To elucidate the effect that the LDL/SCO-spondin complex contained in the eCSF could have on neuroepithelial tissue development, mesencephalon explants from HH20 chick embryos were cultured in the presence of different media (Figure 5): (i) DMEM as a negative control; (ii) Conditioned medium of SCO-explants containing SCO-spondin (conditioned medium with SCO-spondin); (iii) Conditioned medium with SCO-spondin plus antibodies against SCO-spondin; (iv) LDL; (v) Conditioned medium with SCO-spondin plus LDL; and (vi) Conditioned medium with SCO-spondin, LDL and anti-SCO-spondin. Cell proliferation and neuronal differentiation were studied using anti-BrdU and anti-βIII tubulin labeling, respectively. Proliferation and neurodifferentiation were measured by accounting for BrdU immunopositive nuclei and the area immunopositive for tubulin βIII, respectively, in relation to the total area of the explant using the ImageJ program. For this, the total area of the explant was delimited and measured (Figure 6A); the immunopositive area was transformed to binary (Figure 6C), and the number of nuclei or the total area was analyzed by the “Analyze particles” option (Figure 6D).

*Neurodifferentiation* (Figure 6E). As reported before, SCO-spondin (Figure 5B) and LDL (Figure 5G) were able to increase the number of positive tubulin βIII cells several times as compared to explants maintained in DMEM (Figure 5A). On the other hand, the addition of anti-SCO-spondin in the culture medium almost completely inhibited the neurogenic capacity of SCO-spondin (Figure 5C). The most relevant result of this experiment was that the addition of LDL and SCO-spondin at the same time diminished the neurogenic effect that both molecules had separately (Figure 5H). This result suggests that LDL and SCO-spondin interact, and that this interaction inhibits their neurogenic effect. This possibility was reinforced by the observation that the incorporation of SCO-spondin antibodies did not have any effect (Figure 5I), reflecting that SCO-spondin is already inhibited when it forms part of the complex. In the same context, the remnant neurodifferentiation observed in presence of LDL/SCO-spondin (with or without anti-SCO-spondin) might be a consequence of free LDL molecules that do not form part of the complex (Figures 6K,L).

*Cellular proliferation* (Figure 6F). As in previous reports (Vera et al., 2013), SCO-spondin did not generate a significant difference in cellular proliferation as compared to a DMEM control (Figures 5D,E). However, the addition of LDL to the culture medium generated a twofold increase in this parameter (Figure 5J). When both molecules were added together, proliferation was similar to explants maintained in LDL alone (Figure 5H) suggesting that the free LDL molecules were sufficient to exert a proliferative effect. This possibility was sustained by the observation that the addition of anti-SCO-spondin antibodies did not have any effect as compared to explants maintained in LDL alone or in LDL/SCO-spondin (Figures 5J–L, 6F).

## Discussion

Embryonic cerebrospinal fluid is an active signaling center that regulates neuroepithelium development. It contains several morphogens, however, there are no reports about the possible interactions between the different eCSF molecules or how these interactions would modulate their morphogenic roles. The present work showed the interaction between SCO-spondin and LDL, two potent neurogenic molecules present in the eCSF, and how this interaction modulates their neurogenic capacities.

### Bioinformatics Analyses of SCO-Spondin

All members of the LDL receptor family present a cluster of LDLrA domains repeated several times, a condition that is necessary and sufficient to bind LDL (Russell et al., 1989). This domain has a length of 35–40 amino acids and is characterized by the presence of six cysteines responsible for conformation and a DxSDE motif responsible for ligand binding. In this study, a detailed analysis of the LDLrA domains present in SCO-spondin revealed that this protein contained all of the features necessary to bind LDL, since it had a cluster of eight LDLrA domains, each one with six cysteines, and a highly conserved ligand binding motif (Figure 1A). Moreover, these structures were conserved not only between the different LDLrAs of chick SCO-spondin, but also in the SCO-spondin of the different species analyzed (Figure 1B). Conformational analysis of the SCO-spondin fragment containing the LDLrA domains revealed similarity with the structure reported for human LDLr (Rudenko and Deisenhofer, 2003), with these domains surrounding a central pocket to which DxSDE negative motifs are exposed. Structural analysis in human LDLr revealed that this pocket is occupied by the ligand that contains positively charged amino acids on the surface (Hussain et al., 1999; Rudenko and Deisenhofer, 2003; Huang et al., 2010a).

Protein searches in the ExPASy PROSITE protein domain database revealed relatively few proteins containing this domain, and those found were principally proteins of the complement system or members of the LDL receptor family. Surprisingly, besides SCO-spondin, there is only one secreted protein that contains a cluster of this domain; a *Drosophila* mosaic protein called Nudel that has been reported crucial for early development since its inhibition generates the dorsalization of *Drosophila* embryos (Hong and Hashimoto, 1995; LeMosy et al., 2000). However, the LDL binding capacity of Nudel has not been studied, and there is no information on the functions of the eleven LDLrA domains that it contains.

In summary the bioinformatics analyses revealed that SCO-spondin, at least in theory, possesses the elements necessary to be a LDL binding protein.

### Physical Interaction Between LDL and SCO-Spondin

A physical interaction between the SCO-spondin and LDL present in the eCSF was suggested by the identical migration pattern of both molecules in native agarose gel (Figure 2), a possibility reinforced by the immunoprecipitation of LDL with SCO-spondin antibodies (Figure 3). To our knowledge, this is the first report showing the binding of LDL to a soluble carrier molecule.

Immunohistochemical analysis of ApoB and SCO-spondin in the embryonic mesencephalon revealed that both molecules colocalize in the apical membrane of neuroepithelial cells and in eCSF particles (Figure 4). The presence of particles containing lipoproteins was previously reported (Bachy et al., 2008). These authors described the presence of lipoproteins positive for ApoA1 in the eCSF of four-day-old chick embryos, and although ApoA1 mRNA cannot be detected in the brain, the authors found immunoreactivity in the apical region of neuroepithelial cells. These results suggest that extrinsic lipoproteins travel in the eCSF, probably as particles, and enter neuroepithelial cells through the apical membrane in contact with the eCSF. The present immunohistochemical data support this idea and shows that eCSF particles contain SCO-spondin.

### Functional Interaction Between LDL and SCO-Spondin

The functional interaction between SCO-spondin and LDL binding was studied by adding these molecules to the culture medium of mesencephalic explants. In these assays, the formation of LDL/SCO-spondin complex diminished the neurogenic effect of both molecules, but not their proliferative influence (Figures 5, 6E,F). This result is highly relevant since both molecules have a potent effect on neuroepithelial development, and there are currently no reports about regulatory mechanisms.

As was expected, the presence of SCO-spondin or LDL increased neurogenesis in mesencephalic explants. However, an unexpected result was obtained when both molecules were included simultaneously, with neurogenic activity decreasing below the activity found for each molecule separately. This result opens the possibility that SCO-spondin may be binding and transporting LDL when this molecule is highly concentrated and releasing it back to the eCSF when and/or where LDL concentration is low. A similar “buffering” or sequestering function for SCO-spondin was described *in vivo* at the adult stage (Caprile et al., 2003), showing that SCO–spondin participates in the regulation of monoamines CSF concentration. This regulation may occur either by binding and transporting monoamines away or by transiently binding and releasing them back to the CSF, thereby maintaining a constant monoamines concentration (Caprile et al., 2003). In this case, the binding capacity of SCO-spondin monoamines seems to require the TSR domain, since it has sequence similarity with different monoamine transporters (Rodríguez and Caprile, 2001).

The possible function of SCO-spondin as an LDL carrier becomes more relevance when taking into account that the majority of eCSF molecules, LDL included, originate in the plasma (Gato et al., 2004; Parvas et al., 2008). At early stages of development, the transfer of plasma molecules to the eCSF takes place through specific perineural blood vessels located in the ventral mesencephalon and the most anterior part of the ventral prosencephalon, lateral to the floor plate (Parvas et al., 2008). The flow of plasma LDL to the eCSF at this location should generate high local LDL concentration. In this region the brain cavities narrow (future Sylvian aqueduct), and the caudal diencephalic roof plate (where SCO-spondin is secreted) faces the point of entry for plasma molecules, an element that would facilitate binding to SCO-spondin.

For future investigations, it will be interesting to perform similar *in vitro* experiments with different concentrations of LDL and SCO-spondin in order to study affinity in comparison with other members of the LDL receptor family, in addition to researching the capacity of SCO-spondin to bind and release LDL.

### SCO-Spondin, a Carrier of Morphogens in the eCSF?

The binding of SCO-spondin to LDL showed in the present work opens new questions about possible role of SCO-spondin as a morphogen carrier. This possibility is supported by different aspects. Firstly, LDL can carry morphogens of the Hh and Wnt families (Panáková et al., 2005; Willnow et al., 2007). The association between SCO-spondin and lipoprotein-morphogens might have several advantages, such as morphogen mobilization around brain cavities or allowing for the presence of multiple copies of Wnt or Hh on the same lipoprotein particle. These multiple copies would in turn generate a multivalent ligand complex that might be able to promote a homomeric clustering of their respective cognate receptors, as well as heterodimeric interactions between different morphogens (Willnow et al., 2007).

Secondly, LDLrA domains are characterized by the high number of ligands they can bind (Hussain et al., 1999; Strickland et al., 2002). The high versatility of this protein family resides in the flexibility reported for the linker region between the different LDLrA domains that allow them to adapt to different ligands. Likewise, binding occurs not only to a wide range of apolipoproteins present in different lipoproteins, but also to extracellular glycoproteins, such as thrombospondin-1, F-spondin or reelin (Divekar et al., 2014). In this regard, the present investigation did not study the binding of SCO-spondin to other ligands, but this is a possibility that should be explored.

Finally, SCO-spondin is a giant protein of more than 5000 aminoacids, with a modular structure composed by the LDLrA domains described, 26 TSR domains, and one EGF-like domain, among others. The presence of other domains in SCO-spondin, such as the TSR domains that interact with integrins (Caprile et al., 2009) and bind and activate TGF-β (Yee et al., 2004; Young and Murphy-Ullrich, 2004), increase the level of possible interactions and the complexity of this protein’s function. For future investigations on the role of SCO-spondin in the differentiation of neuroepithelial cells, it would be interesting to characterize the different isoforms of SCO-spondin present in this fluid and the interaction of these isoforms not only with LDL but also with other eCSF molecules.

Taken together, these data prompt us to conclude that eCSF is not the sum of molecules with independent effects. In contrast, as it occurs within the ECM, it facilitates the interaction between different molecules and may serve as a reservoir for and distributor of different morphogens.

## Conflict of Interest Statement

The authors declare that the research was conducted in the absence of any commercial or financial relationships that could be construed as a potential conflict of interest.

## Footnotes

^1^http://www.expasy.org/tools/

^2^http://mordred.bioc.cam.ac.uk/~rapper/rampage.php

^3^http://www.pymol.org
